# Relationship between the Prenatal Diagnosis of Placenta Acreta Spectrum and Lower Use of Blood Components

**DOI:** 10.1055/s-0042-1758712

**Published:** 2022-12-29

**Authors:** Néstor Pavón-Gomez, Rita López, Luis Altamirano, Sugey Bravo Cabrera, Gusmara Porras Rosales, Sergio Chamorro, Karen González, Amparo Morales, Juliana Maya, Stiven Sinisterra, Albaro José Nieto-Calvache

**Affiliations:** 1 Hospital Bertha Calderón Roque, Managua, Nicaragua; 2 Latin American Group for the Study of Placenta Accreta Spectrum, Cali, Colombia; 3 Programa de Medicina, Facultad de Ciencias de la Salud, Universidad Icesi, Cali, Colombia; 4 Centro de Investigaciones Clínicas, Fundación Valle del Lili, Cali, Colombia; 5 Clínica de Espectro de Acretismo Placentario, Fundación Valle del Lili, Cali, Colombia

**Keywords:** placenta accreta, prenatal ultrasonographic diagnosis, surgical procedure, blood transfusion

## Abstract

**Objective** To describe the clinical results of patients admitted and managed as cases of placenta accreta spectrum (PAS) at a Central American public hospital and the influence of the prenatal diagnosis on the condition.

**Materials and Methods** A retrospective analysis of PAS patients treated at Hospital Bertha Calderón Roque, in Managua, Nicaragua, between June 2017 and September 2021. The diagnostic criteria used were those of the International Federation of Gynecology and Obstetrics (Fédération Internationale de Gynécologie et d'Obstétrique, FIGO, in French). The population was divided into patients with a prenatal ultrasonographic diagnosis of PAS (group 1) and those whose the diagnosis of PAS was established at the time of the caesarean section (group 2).

**Results:** During the search, we found 103 cases with a histological and/or clinical diagnosis of PAS; groups 1 and 2 were composed of 51 and 52 patients respectively. Regarding the clinical results of both groups, the patients in group 1 presented a lower frequency of transfusions (56.9% versus 96.1% in group 2), use of a lower number of red blood cell units (RBCUs) among those undergoing transfusions (median: 1; interquartile range: [IQR]: 0–4 versus median: 3; [IQR]: 2–4] in group 2), and lower frequency of 4 or more RBCU transfusions (29.4% versus 46.1% in group 2). Group 1 also exhibited a non-significant trend toward a lower volume of blood loss (1,000 mL [IQR]: 750–2,000 mL versus 1,500 mL [IQR]: 1,200–1,800 mL in group 2), and lower requirement of pelvic packing (1.9% versus 7.7% in group 2).

**Conclusion** Establishing a prenatal diagnosis of PAS is related to a lower frequency of transfusions. We observed a high frequency of prenatal diagnostic failures of PAS. It is a priority to improve prenatal detection of this disease.

## Introduction

Placenta accreta spectrum (PAS) is a condition associated to massive hemorrhage and polytransfusion, 1 and patients should be cared for by interdisciplinary groups in experienced centers. 2 3 4 However, the participation of these expert groups relies on a prenatal diagnosis that enables the patient to be guided towards this type of care. The frequency of cases of PAS not diagnosed before laparotomy is variable, but it can be as high as 50%. 5 6 In Nicaragua, among the factors that contribute to the low rate of prenatal diagnoses are the difficulties in training to identify PAS, the absence of centers with a high influx of patients, and the lack of feedback between the centers that carry out the diagnosis and those who deliver treatment. The present work describes the clinical results of the PAS patients managed at a Central American public hospital and the importance of establishing a prenatal diagnosis.

## Materials and Methods

A retrospective analysis of medical records was carried out in search for patients with PAS treated at Hospital Bertha Calderón Roque, in Managua, Nicaragua, between June 2017 and December 2021. The diagnostic criteria used was those of the International Federation of Gynecology and Obstetrics (Fédération Internationale de Gynécologie et d'Obstétrique, FIGO, in French). 7 The population was divided into patients with a prenatal PAS diagnosis by ultrasound submitted to surgery for that reason (group 1) and patients in whom PAS was only detected at the time of the caesarean section (group 2). During this period, the management protocol was standard, with no variations. All patients with a diagnosis of PAS underwent cesarean section at 35 weeks, with a plan for total hysterectomy after extraction of the fetus through the uterine fundus. Specific vascular control strategies nor ureteral catheters were used. All patients included had placenta previa and underwent cesarean hysterectomy, same surgical technique was applied. The present retrospective study was approved by the Institutional Review Board/Ethics Committee for Biomedical Research (under no. 1494). A descriptive statistical analysis was carried out; The continuous variables were expressed as median and interquartile range (IQR) values, and they were analyzed using the Mann-Whitney U test. The qualitative variables were expressed as absolute and relative frequencies, and the comparison between them was made using the Chi-squared test or the Fisher exact test according to the case. Statistical significance was defined as *p* < 0.05. The analyses were performed using the STATA (StataCorp LLC, College Station, TX, United States) software, version 14.

## Results

During the study period, 114 women with a histological and/or clinical diagnosis of PAS were found: 51 patients had a prenatal PAS diagnosis by ultrasound (group 1), and 63 were only diagnosed when they underwent laparotomy (group 2).

Chart 1 summarizes the clinical results of both groups, showing a lower frequency of transfusions in group 1 (56.9% versus 87.3% in group 2), as well as the use of a lower number of red blood cell units (RBCUs) in said transfused patients (median: 1; IQR: 0–4 versus median: 3; IQR: 2–4 in group 2). The frequency of 4 or more RBCU transfusions was also lower in group 1 (29.4% versus 44.4% in group 2). Group 2 underwent surgery at a higher gestational age (mean: 38 weeks; IQR: 35–39 weeks versus median: 34 weeks; IQR: 32–36 weeks in group 1), with a lower rate of participation of interdisciplinary groups (62.4% versus 90.2% in group 1) and of elective surgeries than group 1 (22.2% in group 2 versus 78.4% in group 1). Group 1 also exhibited a non-significant trend toward a lower volume of blood loss (median: 1,000 mL; IQR: 750–2,000 mL versus median: 1,500 mL; IQR: 1,300–2,200 mL in group 2), lower requirement of pelvic packing with compresses to control bleeding (1.9% versus 7.9% in group 2), surgical reinterventions (11.8% versus 17.5% in group 2), and surgical site infection (1.9% versus 4.8% in group 2) than group 2. In a high percentage of patients (35; 30.9%), the histological diagnosis was not available because the tissue had not been not processed by the pathology department.

**Chart 1:** Comparison of the clinical results of PAS patients with and without a prenatal diagnosis

		Group 1 (n = 51): WITH prenatal diagnosis	Group 2 (n = 63): WITHOUT prenatal diagnosis	*p* -value
Gestational age at surgery (in weeks)*		34 (32-36)	38 (35-39)	0.003
Surgical time (in minutes)*		103 (82-145)	94 (74-129)	0.235
Interdisciplinary group participation: n (%)		46 (90.2)	33 (62.4)	0.003
Elective surgery: n (%)		40 (78.4)	14 (22.2)	0.002
Bleeding volume (mL)*		1000 (750-2000)	1500 (1300-2200)	0.347
Transfusions: n (%)		29 (56.9)	55 (87.3)	0.005
Number of RBCUs transfused		1 (0-4)	3 (2-4)	0.027
4 or more RBCUs: n (%)		15 (29.4)	28 (44.4)	0.039
Bladder injury: n (%)		7 (13.7)	9 (14.2)	0.923
Ureteral injury: n (%)		2 (3.9)	0	−
Urinary fistula: n (%)		0	1 (1.6)	−
Pelvic packing with compresses: n (%)		1 (1.9)	5 (7.9)	0.380
Reintervention: n (%)		6 (11.8)	11 (17.5)	0.996
Wound infection: n (%)		1 (1.9)	3 (4.8)	0.666
Death: n (%)		1 (1.9)	1 (1.6)	0.104
Histological analysis: n (%)	Placenta acreta	24 (47.1)	34 (53.9)	0.890
	Placenta increta	11 (21.6)	6 (9.5)	
	Placenta percreta	2 (3.9)	2 (3.2)	
	No histological study	14 (27.4)	21 (33.3)	

Abbreviations: PAS, placenta accreta spectrum; RBCU, red blood cells unit.

Note: *Median (interquartile range).

## Discussion

Less than half of our cases (44.7%) had a prenatal PAS diagnosis (group 1). Patients in group 1 had a lower frequency of transfusions and those among them who received blood components required a lower number of RBCUs. Although some expert groups have reported excellent performance of the PAS ultrasonographic diagnosis, 8 9 even in some high-income countries the frequency of false positives is close to that observed in the Nicaraguan population, with a rate of intraoperative diagnosis close to 50%. 5 6 There are multiple factors that explain a poor performance in establishing a PAS prenatal diagnosis in our population. Although all the patients included in the present study underwent prenatal follow-up visits and periodic ultrasonographic scans, Nicaragua has not established protocols to diagnose PAS. Additionally, there are few maternal-fetal medicine specialists or prenatal ultrasonography experts. Finally, there is no chair in the diagnosis and treatment of PAS in the obstetrics training programs in our country. It is important to point out these difficulties as the first step towards improving the prenatal identification of PAS. It is likely that the knowledge of a prenatal PAS diagnosis in group 1 facilitated the scheduling of the surgical procedure, which was elective in 78.4% of these patients, unlike group 2, in which it was elective in 22.2% of the cases, and at a higher gestational age (median: 34 weeks; IQR: 32–36 weeks in group 1 versus median: 38 weeks; IQR: 35–39 weeks in group 2).

One of the advantages of knowing the PAS diagnosis is the possibility of “scheduling” the participation of the interdisciplinary groups during surgery. 10 11 12 Our hospital does not have an interdisciplinary group dedicated to the treatment of PAS (a “PAS team”); however, patients from group 1 were treated by the more experienced surgeons available, which included the urologist and the teneral surgeon on duty that day. This was possible in 90.2% of the cases in group 1, and only in 62.4% of the cases in group 2. In the event that the diagnosis of PAS was a “surprise” during the laparotomy, calling the surgeon and the urologist on duty was left at the discretion of the obstetricians in charge of the surgery. Other authors 5 have pointed out the importance of prenatal diagnosis and its relationship with lower levels of blood loss and lower use of transfusions, and they also coincide in documenting important differences in the management of patients with and without a prenatal diagnosis.

Our hospital is a reference center for the most critical obstetric conditions in the country, but like many other hospitals with similar characteristics, it does not have a PAS team. 13 Our flaws in the prenatal diagnosis (intraoperative finding of PAS in 55.2% of our cases) and histological analysis (absence of analysis by a pathologist in 35 cases) result in an opportunity to improve the quality of care in our center.

The present study has limitations. Its retrospective design makes it more susceptible to bias. The absence of histological confirmation in 30.7% of the cases enabled the inclusion of non-PAS cases; however, patients whose medical record described macroscopic findings compatible with the FIGO definition were included. The present is the first evaluation of the clinical results of PAS management in Nicaragua, and one of the few that have been carried out in Central America.

The results shed light on the need to design improvement plans at the national level, with the need for multicenter prospective studies to confirm our observations and evaluate the effect of interventions already implemented, such as the creation of a PAS team that is provided with specific training in the management of this disease, the proposal of including PAS screening in routine prenatal care appointments for women with risk factors, and the project of including a section about PAS in the national guidelines for the treatment of postpartum hemorrhage.

## Conclusion

The presence of a prenatal diagnosis of PAS is related to a lower frequency of RBCU transfusions. We observed a high frequency of failures within the prenatal PAS diagnostic steps. It is a priority to improve the prenatal detection of this disease.
